# Synthesis and Regeneration of A MXene-Based Pollutant Adsorbent by Mechanochemical Methods

**DOI:** 10.3390/molecules24132478

**Published:** 2019-07-05

**Authors:** Mohammadtaghi Vakili, Giovanni Cagnetta, Jun Huang, Gang Yu, Jing Yuan

**Affiliations:** 1 Green Intelligence Environmental School, Yangtze Normal University, Chongqing 408100, China; 2 State Key Joint Laboratory of Environment Simulation and Pollution Control (SKJLESPC), Beijing Key Laboratory of Emerging Organic Contaminants Control (BKLEOCC), Key Laboratory of Solid Waste Management and Environment Safety, School of Environment, POPs Research Center, Tsinghua University, Beijing 100084, China

**Keywords:** MXene, mechanochemical synthesis, high energy ball milling, adsorption

## Abstract

In the present study, an adsorbent material for removal of organic contaminants in wastewater is synthetized by a green and facile mechanochemical method. It is composed of Ti_3_C_2_T_x_ MXene layers (obtained by mechanochemical etching of MAX phase with concentrated HF) pillared with terephthalate by rapid direct reaction. Such material shows high specific surface area (135.7 m^2^ g^−1^) and excellent adsorption capability of methylene blue (209 mg g^−1^) because of the larger interlayer space among MXene sheets and free carboxylate groups of terephthalate. The spent adsorbent is reutilized (with addition of sole aluminum) to synthetize the MAX phase by mechanochemical procedure, where the terephthalate and the pollutant are carbonized into the carbide. In this way, new MXene-based adsorbent can be re-synthetized for further use.

## 1. Introduction

Contaminant removal from wastewater is fundamental to prevent the spread of toxic compounds in the environment, which poses a serious threat to human health. Adsorption is recognized to be one of the most effective technologies that can be implemented to treat industrial wastewater [1]. It is cheap, effective, easy to operate, and produces high-quality effluent, thus it is widely utilized [2]. Additionally, it is a versatile technology, thanks to the large number of adsorbing materials that have been developed to capture organic and inorganic pollutants [3]. Therefore, the type and nature of adsorbing materials influence efficiency of adsorption process. However, regeneration of spent adsorbents requires expensive and environmentally unfriendly methodologies such as direct disposal in landfill, incineration, or washing with solvents, etc., which often cause secondary pollution [4,5].

Mechanochemical (MC) methods have been extensively applied to produce a number of adsorbing materials [6,7,8,9,10]. These methods are generally based on the insertion of great amounts of mechanical energy into materials to achieve unique properties and modifications [11,12,13,14]. High energy ball milling is largely employed to provide such energy to solids through countless hits of milling balls occurring into high speed moving jars [15]. Since solvents are not required (at most, only small amounts), MC synthesis is considered a green way to prepare materials [16,17]. More interestingly, some adsorbents that were used to capture organic pollutants can be also regenerated by simple high energy ball milling [7]. Indeed, organic pollutants undergo mineralization to graphitic and amorphous carbon during high energy milling [18,19]. This fact suggests that carbon-based materials, like carbides, might be easily regenerated by MC methods.

**Figure 1:**
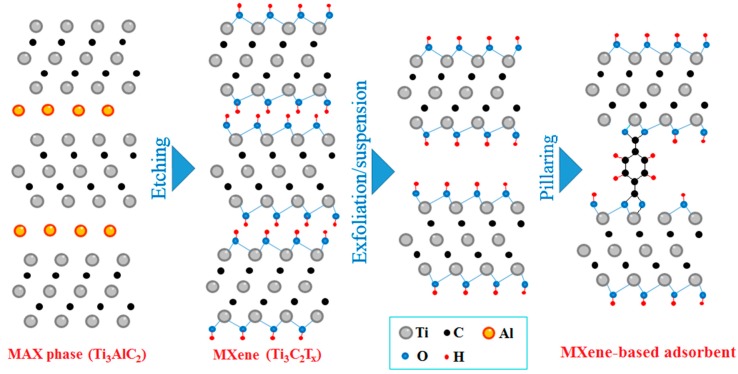
Scheme of MXene sheets preparation process from MAX phase and pillaring approach used in this study to synthetize the MXene-based adsorbent.

MXene are a recently developed class of bidimensional transition metal carbides and nitrides (with sheet-like structure) that can be prepared by etching of special layered carbides and nitrides called MAX phases (with generic formula M_n+1_AX_n_, where M is an early transition metal, e.g., Ti, Mo, V, etc.; A is an A-group element, e.g., Al or Si; and X is C or N) [20,21] (Figure 1). MAX phase carbides can be easily MC synthetized by high energy milling of transition metal dioxide, aluminum, and carbon [22]. Etching of Al atoms to release bidimensional carbide layers is realized by mixing the MAX phase with concentrated hydrofluoric acid for long time (typically, 24 h), and the etching method is responsible for the formation of different functional groups (e.g., ‒F, ‒OH, =O, etc.) on the MXene surface (usually indicated as T_x_) [23]. It can be realized with other etching agents (e.g., LiF + HCl conc., NH_4_F) and exfoliation methodologies (e.g., ultrasonication), but such a process can still be hardly scaled-up in a cost-effective manner due to large consumption of solvent and energy [24,25,26]. Therefore, a MC exfoliation process of the MAX phase has been also investigated by utilizing dimethyl-sulfoxide as solvent [27]. MXenes have peculiar properties such as high conductivity, comparable with that of graphene, which make them good candidates for energy storage and preparation of electrodes; and good biocompatibility, with potential applications in biosensing [28]. It was also proved that MXenes have quite good adsorption capability [29], but this is quite limited by their clayey structure. Specifically, the interlayer surfaces are not easily accessible because of their stacked structure with a small interlayer distance. This can be increased by intercalation of other molecules, like surfactants [30,31] and hydroxides [32].

The aim of this work is to overcome the issue of small interlayer space by introducing pillars among MXene bidimensional layers. In this way, improvement of adsorption capability toward organic pollutants is achieved. In addition, regenerability of the material is investigated. Specifically, organic pillars and pollutants can be retransformed into carbide (i.e., MAX phase) by facile MC processing. Last but not least, a more effective etching methodology is proposed to produce MXenes from MAX phases. It consists of MC exfoliation in presence of reduced volume of concentrated HF.

## 2. Results

### 2.1. Mechanochemical Etching

**Figure 2:**
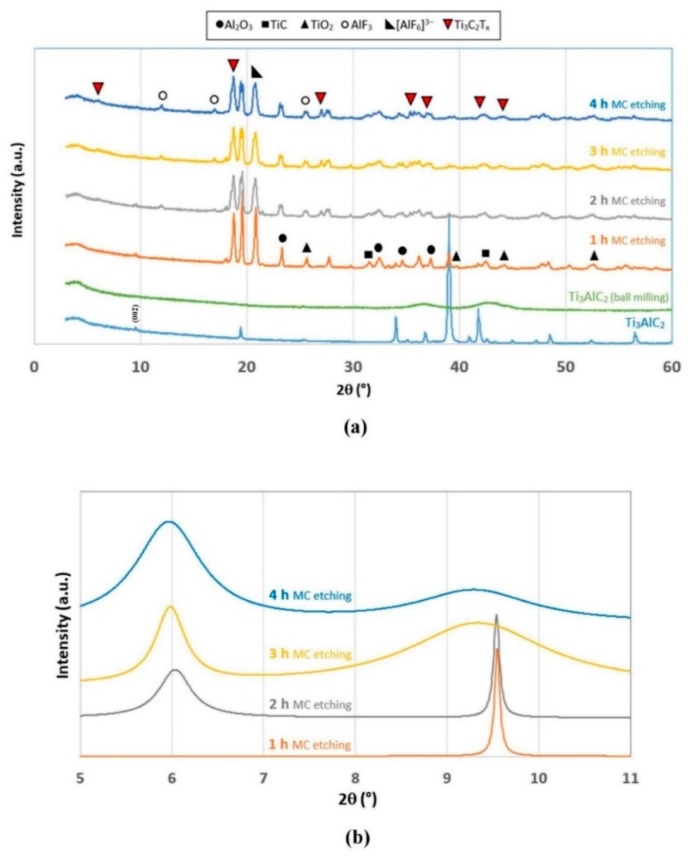
(**a**) Diffractograms of mechanochemical (MC) etching with HF at different milling times. (**b**) Rietveld refinement of XRD peak of (002).

**Figure 3:**
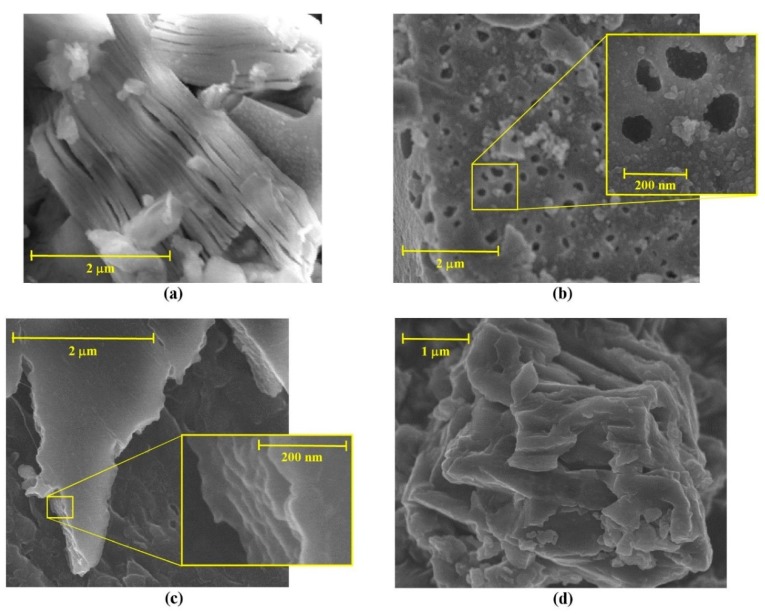
SEM photographs of (**a**) Ti_3_C_2_T_x_ MXene after 4 h MC etching, (**b**) terephthalate-pillared MXene, (**c**) imidazole-pillared MXene, (**d**) oxalate-pillared MXene.

High energy ball milling was utilized to prepare Ti_3_C_2_T_x_ MXene from Ti_3_AlC_2_ MAX phase (which was synthetized from TiO_2_, Al, and graphite by ball milling according to the procedure of Zhu et al. [22]) utilizing a MC etching method with HF. Specifically, 1 g MAX phase powder was exfoliated in solely 20 mL hydrofluoric acid (40% solution) by ball milling. The product was constituted by a green foamy liquid (the Ti_3_C_2_T_x_ suspension) and a black precipitate (mainly aluminum oxide, titanium carbide, and unreacted MAX phase). X-ray diffractograms at different milling time, utilizing moderate energy provision to prevent significant crystal structure demolition, demonstrate that exfoliation occurred (Figure 2a). The MAX phase powder has typical amorphous characteristics, due to the long time milling required to produce it from TiO_2_, carbon, and metallic Al powder. For the sake of comparison, a diffractogram of a commercial Ti_3_AlC_2_ is also provided in Figure 2a; here the most significant peak that at 2θ = 9° corresponds to the (002) crystal plane that is related to interlayer space between Ti_3_C_2_ planes bound by Al^3+^ ions [32]. After 1 h MC etching, peaks corresponding to MAX phase preparation by-products (i.e., Al_2_O_3_ and TiC) and unreacted reagent (i.e., TiO_2_) as well as new products generated by HF etching (i.e., AlF_3_ and an hexafluoroaluminate salt), become visible. During MC etching, the Ti_3_AlC_2_ peaks decrease in intensity, while those of exfoliated carbide (Ti_3_C_2_T_x_) emerge and become stronger. In particular, the 9° peak weakens along with milling time and shifts towards near 6°, which is the interlayer space of the newly formed MXene. This latter has a wider interspace because of intercalation of solvent molecules, thus having a book-like multilayered clayey structure (Figure 3a).

Rietveld refinement of peaks at 9° and 6° at diverse milling times (Figure 2b) clearly demonstrates the dismantling of compact layered MAX phase structure (9° peak intensity reduction and broadening) as MXene builds up (6° peaks intensity increase and broadening). Specifically, MXene interlayer space increases along with milling, as proved by the sensible shift of its corresponding peak toward lower 2θ.

In truth, XRD results indicate an incomplete delamination of Ti_3_AlC_2_ MAX phase after 4 h MC etching, since its peaks are still visible. However, the obtained conversion was sufficient to produce the pillared MXene adsorbent (as explained later), in terms of both conversion and reaction rate. Therefore, no further optimization of the MC etching process was carried out.

### 2.2. Adsorbent Synthesis and Performance

Ti_3_C_2_T_x_ MXene prepared by MC etching was suspended in water. The liquid fraction was separated and mixed with one of the organic ligands that were employed to prepare the adsorbent (i.e., potassium terephthalate, imidazole, and potassium oxalate). All investigated ligands caused a rapid precipitation (<1 min) of the adsorbent by sole gentle mixing with a glass stick; the reaction solution turned from green to brown.

**Figure 4:**
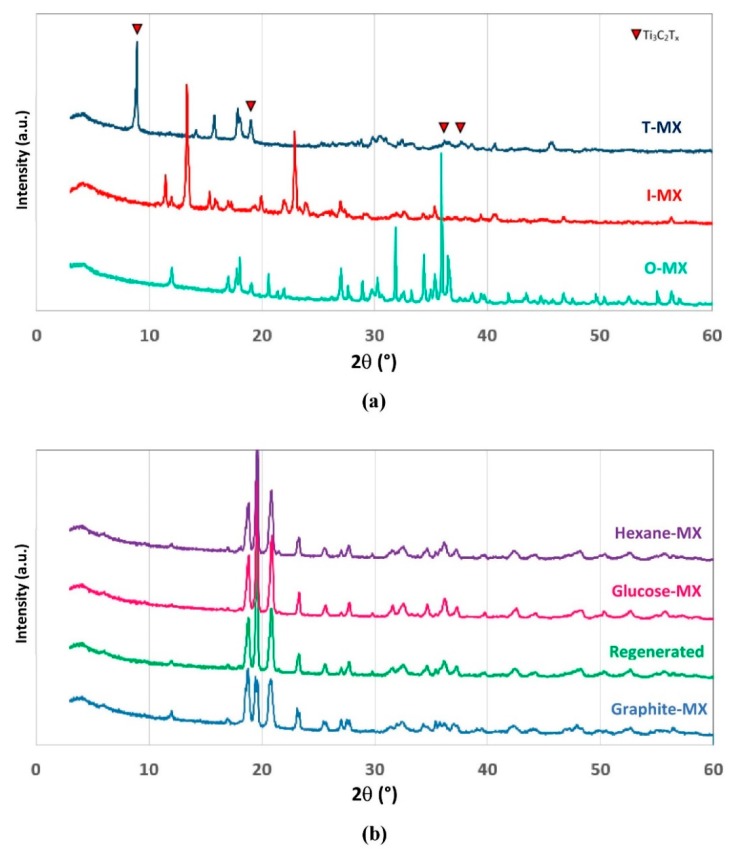
Diffractograms of (**a**) MXene-based adsorbents materials with different ligands, and (**b**) of MXene prepared with graphite, spent T-MX (after methylene blue adsorption) and different precursors.

The so-prepared materials have rather good specific surface areas. Specifically, BET areas were 135.7, 32.6, and 21.8 m^2^ g^−1^ for the Ti_3_C_2_T_x_ MXene bound with terephthalate (T-MX), imidazole (I-MX), and oxalate (O-MX), respectively, compared with the 5.4 m^2^ g^−1^ area of MC etched MXene. SEM photographs (Figure 3b–d) shed light on such discrepancies. T-MX is a multilayered material with clearly visible macropores (~100 nm diameter). I-MX and O-MX maintain a layered structure that is similar to that of the original MXene. It can be hypothesized that terephthalate bound Ti atoms that are largely available on both sides of the sheet-like MXene and are highly reactive [23]. Conceivably, this reaction is similar to that occurring in the generation of metal-organic frameworks, where electron doublets in carboxylate coordinate metal ions form stable crystalline structures [33]. Hence, it can be inferred that the prepared material has a pillared structure with large interlayer space (approximatively equal to terephthalate length). This is corroborated by the high XRD peak at 2θ ≈ 8° (Figure 4a), which highlights a large interlayer space between Ti_3_C_2_T_x_ planes. Peaks at 2θ < 10° are not clearly visible in diffractograms of I-MX and O-MX, thus implying that the multilayered structure might be intercalated by the ligands at most, but certainly they are not stably bound to the MXene sheets as pillars. Intercalation explains the sensibly higher specific surface areas of both materials respect to MXene, but much lower than that of T-MX.

**Figure 5:**
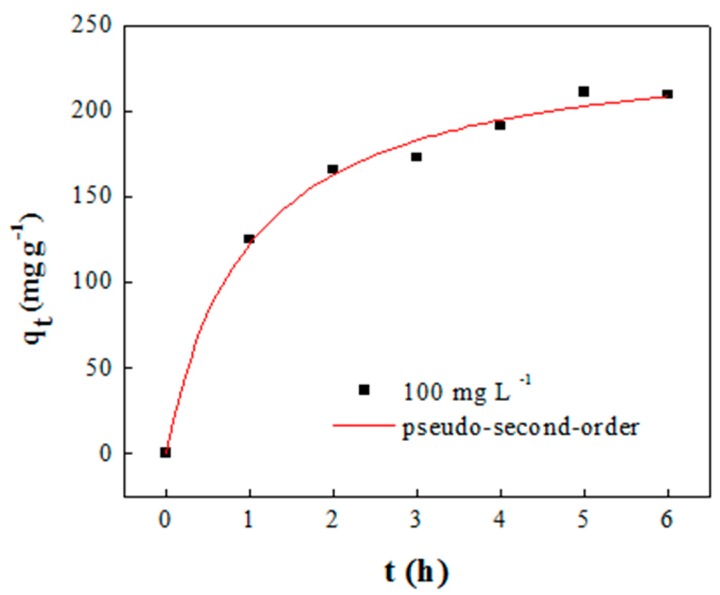
Adsorption kinetic of methylene blue onto T-MX (expressed as the adsorption capacity q_t_ vs contact time t) and best-fitting by the pseudo-second-order model (10 mg T-MX, pH 7, 150 rpm, 20 °C).

Analytical results clearly suggest that T-MX might have good adsorption properties, so it was tested with methylene blue. The adsorption of such dye onto T-MX within the first 2 h of process is fast, while in the late phase it becomes slow until the adsorption equilibrium is reached (5 h) (Figure 5). The initial fast adsorption is ascribable to the presence of high amount of unoccupied and available adsorption sites. However, with the adsorption process ongoing, T-MX surface is increasingly covered by dye molecules and, therefore, accessibility and availability of functional groups decrease, resulting in a reduced dye removal rate [34].

Table 1 shows the equilibrium adsorption capacity and kinetic model constants. The correlation coefficients (R^2^) and the chi squared (χ^2^) of the pseudo-second-order model were calculated to be higher and lower, respectively, than those of the pseudo-first-order model. Moreover, adsorption capacity estimated by the pseudo-second-order model is close to the value found experimentally (209.5 mg g^−1^), corroborating the better agreement of such model with experimental data. This fact suggests that adsorption of methylene blue onto the T-MX is mainly based on chemisorption [35]. Such good performance of T-MX is conceivably due to the increased interlayer space, and possibly to terephthalate carboxylates on the material surface that can bind ionically the methylene blue molecules.

### 2.3. Mechanochemical Regeneration of Spent Adsorbent

Adsorption test with methylene blue demonstrates good adsorption capacity of T-MX, which can be potentially utilized for water reclamation. After adsorption of the dye, the spent material is essentially constituted by titanium and carbon. This latter certainly derives from the carbide Ti_3_C_2_T_x_, but a significant fraction comes from terephthalate pillars and methylene blue. Hence, two out of three components of the MAX phase are still present in the spent adsorbent. Such used-up material, mixed with an appropriate amount of Al powder, was utilized to regenerate the MAX phase by MC synthesis, and then MC etched to re-obtain the MXene. XRD pattern of the regenerated Ti_3_C_2_T_x_ (Figure 4b) is similar to that of the MXene produced from MAX phase prepared with graphite. This result proves that T-MX can be regenerated by MC procedure (passing by MC etching) an indefinite number of times. The pillars and the pollutant are carbonized by high energy milling, according to the well-known phenomenon of organic compound carbonization [36]. Then, the generated carbon is reduced (thanks to Al) and incorporated into the Ti_3_AlC_2_ structure.

Supposedly, T-MX can be used to adsorb various organic cationic pollutants (e.g., other cationic dyes, some antibiotics, etc.). In order to assess the regenerability of the material with various organics, an experiment was carried out. The MAX phase was prepared with diverse carbon precursors (i.e., hexane and glucose) instead of graphite to demonstrate that diverse carbonaceous compounds can be viably incorporated in the carbide structure of the MAX phase. Once again, it was verified by XRD that MXene can be prepared from the so-synthetized MAX phases as well (Figure 4b). Such a result indicates that the carbon precursor is relatively unimportant for MC synthesis of MAX phase. Hence, theoretically any kind of organic pollutant can be carbonized and incorporated, allowing recycle of the spent adsorbent.

## 3. Discussion

In this work, a facile method for the synthesis of an adsorbent with high adsorption capacity is proposed. The material is constituted by Ti_3_C_2_T_x_ MXene pillared with terephthalate. MXene were prepared by rapid MC etching with a small volume of concentrated HF. In this sense, this methodology is greener than usual ones. MC exfoliation in organic solvent has been previously reported [27]. However, it is known that the etching process can influence the functional group composition on MXene surface [23]. The majority of the MXene and MXene-based materials investigated to date are realized by HF etching, and it can be inferred that the required properties are partly due to the presence of F atoms on their surfaces. Therefore, the MC etching method proposed in the present study can be considered for potential scale-up of the approach commonly used in the laboratory. Nonetheless, it should be noted that possible contamination may derive from iron and alloying elements of high energy mill tools (i.e., balls and jar). Leaching of such elements in the reaction solution is very plausible, owing to the strong acidic conditions. They likely remain in solution as fluorides, but it cannot be excluded that the very reactive Ti atoms on MXene sheet surfaces might bind them. This aspect needs further investigation in future works.

The T-MX adsorbent showed very good performance, thanks to the increased interlayer distance. Other authors have already tried to enhance adsorption capability of MXene towards methylene blue by intercalating hydroxides (i.e., LiOH or NaOH) [29], actually obtaining improved uptake capacity (189 mg g^−1^) [32]. It can be inferred that the better performance of T-MX is mainly due to the larger interlayer space achieved by pillaring, which facilitates diffusion of the dye. Nevertheless, it cannot be excluded that free terephthalate carboxyl groups might also contribute to adsorption capability.

Results of MAX phase MC synthesis form spent T-MX adsorbent or other carbon precursors clearly demonstrate the regenerability of such material, independently from the adsorbed organic pollutant. In fact, the pollutant and the tested carbon precursors are significantly carbonized, allowing the reformation of the carbide. The sole material input in this process is Al powder, while the other elements (Ti and C) are substantially reutilized. Theoretically, Al can be recovered by electrochemical reduction from the etching solution (where it is in the form of fluoride salts). But, this would require adequate experimentation.

In conclusion, MC methods were used to prepare and regenerate a MXene-based adsorbent with enhanced adsorption capacity. Such a material can be potentially utilized to treat various pollutants in wastewater, and can be regenerated easily by green MC synthesis process.

## 4. Materials and Methods

### 4.1. Materials

Titanium dioxide, aluminum powder, graphite, and glucose (Yong Da Chemicals Ltd., Jinan, China) were used for the MC synthesis of MAX phases, together with hexane (J.T. Baker Inc., Phillipsburg, NJ, USA). Hydrofluoric acid 40% aqueous solution (Beijing Chemical Works Ltd., Beijing, China) was employed to execute MC etching experiments. Potassium terephthalate, imidazole, and potassium oxalate (Merck KGaA, Darmstadt, Germany) were utilized for preparation of adsorbent materials. Adsorption test was carried out employing methylene blue (Shanghai Macklin Biochemicals Ltd., Shanghai, China) as pollutant. Deionized water was prepared by a Milli-Q system (Millipore, Milwaukee, WI, USA).

### 4.2. Mechanochemical Synthesis of Ti_3_AlC_2_ MAX Phase

Ti_3_AlC_2_ MAX phase was synthetized according to a modified version of the method described by Zhu et al. [22]. Titanium oxide, aluminum powder, and graphite were used to prepare Ti_3_AlC_2_ by the reaction:

3 TiO_2_ + 5 Al + 2 C → Ti_3_AlC_2_ + 2Al_2_O_3_

Amounts of 3.01 g TiO_2_, 1.69 g Al, and 0.30 g graphite (corresponding to TiO_2_:Al:C molar ratio of 3:5:2, and a total mass of 5 g) were put into a 75 cm^3^ mill jar with 25 stainless steel balls (Ø10 mm, total ball mass of 100 g). The powder mixture was milled utilizing a planetary ball mill (Pulverisette 4, Fritsch GmbH, Germany) at 300 rpm for 10 h.

A similar procedure was adopted for regeneration of the spent adsorbent (3.31 g), which was mixed with Al powder (1.69 g) and milled under the same conditions mentioned above. With regard to the MC synthesis of MAX phase with different carbon precursors, hexane and glucose were mixed with adequate amounts of TiO_2_ and Al, keeping the same molar ratio and reagent total mass. Specifically, 0.55 mL (0.36 g) hexane was mixed with 2.97 g TiO_2_ and 1.67 g Al; 0.69 g glucose was added to 2.76 g TiO_2_ and 1.55 g Al. Milling parameters of the MC synthesis were the same described above.

### 4.3. Mechanochemical Etching of Ti_3_AlC_2_ MAX Phase for Ti_3_C_2_T_x_ MXene Synthesis

MC etching was executed with another planetary ball mill (QM-3SP2, Nanjing University Instrument Corporation, Nanjing, China), which is less energy intensive than Pulverisette 4 (according to calculations done previously [37]). In this way, it was possible to mostly preserve the structure of exfoliated MXene. The Ti_3_AlC_2_ MAX phase (1 g) was placed in a 40 cm^3^ mill jar with 20 mL HF 40% and 60 g stainless steel balls (Ø5 mm). Then, the mixture was milled at 200 rpm for various time extents. The milled material was washed with deionized water by several cycles of suspension and centrifugation, until the water pH was higher than 5. Subsequently, the MXene were dried and stored under vacuum at room temperature to prevent its excessive oxidation.

### 4.4. Adsorbent Preparation

MXene powder (~5 g) was re-suspended in 100 mL deionized water and subjected to brief (~10 min) ultrasonication to accelerate delamination of the material. An amount of 50 mL suspension (paying attention to avoid withdrawing solids) was mixed with 50 mL 0.5 M aqueous solution of each ligand (i.e., terephthalate, imidazole, and oxalate). The precipitate was separated by centrifugation, washed with deionized water, and centrifuged again to remove water. The so-prepared adsorbents were dried in air at room temperature.

### 4.5. Adsorption of Methylene Blue onto Terephthalate-MXene

The adsorption capability of terephthalate-Mexene material was studied by assessing adsorption kinetics of methylene blue. Adsorption experiment was conducted by placing 10 mg T-MX adsorbent in a 100 mL Erlenmeyer flask with 50 mL of methylene blue solution (100 mg L^−1^). The experiment was repeated with various flasks that were agitated on an orbital shaker for different contact times varying from 5 to 360 min at pH 7, 150 rpm at room temperature (~20 °C). Methylene blue concentration was quantified utilizing a spectrophotometer (DR 5000, Hach Lange, Germany) to measure absorption at λ = 660 nm; samples were pre-filtered by a nylon syringe filter before analysis. The amount of adsorbed methylene blue by the T-MX (*q_t_*) at time t was measured using Equation (1). The pseudo-first order and pseudo-second order kinetic models were used to study the adsorption kinetic [38].

(1) $$q_{t}=V\left(C_{0}-C_{t}\right)/m$$

where *V* is the volume of methylene blue solution (L), *C*_0_ is the initial concentration of methylene blue (mg L^−1^), *C_t_* is the concentration of methylene blue (mg L^−1^) at time of *t*, and *m* is the dry weight of T-MX (g).

### 4.6. Characterization of the Materials

The synthetized materials were characterized by XRD using a diffractometer (Smartlab, Rigaku, Japan) from 2θ = 3° to 60° and a scanning rate of 10° min^−1^. In addition, SEM photographs were taken by a LEO-1530 microscope (LEO, Germany) for morphological investigation.

## 5. Conclusions

The present study was aimed to the preparation of a material with enhanced adsorbing capacity toward organic pollutants. The adsorbent is composed by MXene sheets pillared by terephthalate and showed remarkable specific surface area and adsorption capacity (employing methylene blue as a model pollutant). It was synthetized by a novel MC etching method using a high energy ball mill to exfoliate a titanium MAX phase with a limited volume of concentrated hydrofluoric acid. Pillaring procedure was realized by simple mixing of MC etched MXene suspension with pillar solution (i.e., therephtalate). It was also verified that the spent adsorbent (after adsorption) can reform the MAX phase by MC synthesis and adding only aluminum powder, thanks to the carbonization of pillars and pollutants into carbide. Afterwards, it was proved that the MXene produced from the so-prepared MAX phase was still usable for adsorbent synthesis. Hence, such recyclable material can be used an indefinite number of times to eliminate organic pollutants from wastewater and ensure their safe destruction.

## Figures and Tables

**Table 1:** Kinetic parameters of kinetic model for adsorption of methylene blue on T-MX.

Adsorption Capacity (mg g^−1^)	Pseudo-First-Order *				Pseudo-Second-Order **			
**209.5**	***q_e_***	***K*_1_**	**R^2^**	**χ^2^**	***q_e_***	***v*_0_**	**R^2^**	**χ^2^**
	242.89	0.837	0.982	95.35	205.60	246.77	0.993	38.58

* Pseudo-first-order: $q_{t}=q_{e}\left(1-e^{-K_{1}t}\right)$; ** Pseudo-second-order model: $\frac{t}{q_{t}}=\frac{1}{k_{2}q_{e}^{2}}+\frac{t}{q_{e}}=\frac{1}{v_{0}}+\frac{t}{q_{e}}$.
